# Evaluating and shaping early childhood policy in New Mexico: using kindergarteners’ developmental health as a population health measure

**DOI:** 10.1186/s12889-026-27196-5

**Published:** 2026-04-02

**Authors:** Judith L. Perrigo, Efren Aguilar, Joshua L. Bader, Jordan Morales, Shirley Russ, Neal Halfon

**Affiliations:** 1 Department of Social Welfare, University of California, Los Angeles (UCLA) Luskin School of Public Affairs, 337 Charles E. Young Dr. E, Los Angeles, CA 90095 USA; 2 UCLA Center for Healthier Children, Families & Communities, 10990 Wilshire Blvd., Suite 260, Los Angeles, CA 90024 USA; 3 Department of Medicine, UCLA David Geffen School of Medicine, 10833 Le Conte Ave., Los Angeles, CA 90095 USA; 4 Department of Health Policy and Management, UCLA Fielding School of Public Health, 650 Charles E Young Dr S, Los Angeles, CA 90095 USA

**Keywords:** Early Development Instrument (EDI), National Neighborhood Equity Index (NNEI), Prevention and Early Intervention (PEI), New Mexico, Population health, Policy reform

## Abstract

**Background:**

New Mexico is the first state in the U.S. to implement a comprehensive, statewide assessment of children’s developmental health in tandem with sweeping early childhood policy reforms. These reforms—including universal child care and preschool—aim to strengthen the early childhood ecosystem and improve population health. Yet, little is known about the developmental health of kindergarteners in the state or how demographic and neighborhood contexts shape readiness at school entry. This study provides the first population-level snapshot of developmental health among New Mexico’s kindergarteners during a period of significant policy transformation.

**Methods:**

We conducted a statewide cross-sectional study of kindergarteners assessed from April to May 2024. Data represents 116 school districts, 441 schools, 600 neighborhoods, and 1,380 classrooms (*N* = 18,974 children). Children’s developmental health and school readiness was measured using the Early Development Instrument (EDI), which assesses five domains: physical health and well-being, social competence, emotional maturity, language and cognitive development, and communication skills and general knowledge. Neighborhood context was assessed using the National Neighborhood Equity Index (NNEI), which classifies neighborhoods into four levels (zero, low, medium, high inequity). Descriptive statistics were used to examine child characteristics, developmental outcomes, and neighborhood patterns. Multilevel regression models examined associations between EDI outcomes and child characteristics (sex, ethnorace, English Language Learner [ELL] status, and Individualized Education Program [IEP] status) and neighborhood context (NNEI).

**Results:**

Children had a mean age of 6.21 years with a 0.33 standard deviation, and 51% were boys. More than 70% of kindergarteners were overall on-track in developmental expectations across individual domains; however, fewer than 48% met expectations across all five domains simultaneously. Vulnerabilities were more pronounced among boys, African American/Black and American Indian/Alaska Native children, ELLs, and students with an IEP. 61% of children resided in neighborhoods with zero or low equity barriers. A clear social gradient was observed, with 23% of children in neighborhoods with NNEI level ‘zero’ demonstrated overall vulnerability in ≥ 1 domain, compared with 33% of children in neighborhoods with NNEI level ‘high.’

**Conclusions:**

Findings indicate generally positive developmental health among New Mexico kindergarteners, yet significant disparities persist across demographic and neighborhood lines. The integration of bold policy reforms with a statewide developmental assessment represents a promising model for monitoring population health and identifying opportunities for targeted early childhood prevention and early intervention supports.

## Introduction

Early childhood is a critical window in which rapid developmental changes create both heightened vulnerability and remarkable opportunity [1–3]. These early experiences—whether adverse or enriching—become biologically embedded, influencing cognitive, emotional, and behavioral regulation and forming the foundation for lifelong health. Extensive evidence links these early experiences to long-term outcomes [4, 5], including increased risk of chronic conditions such as cardiovascular disease [6] and depression [7]. An important yet often underappreciated component of early experience is the neighborhood context in which children develop. Early brain development is shaped not only by caregiving relationships but also by broader environmental conditions that structure children’s daily exposures, including neighborhood safety, collective efficacy, and economic conditions [8, 9]. As a result, neighborhoods operate as upstream determinants of early developmental health, with enduring implications across the lifespan.

A growing body of research documents substantial disparities in early childhood developmental health across sociodemographic and neighborhood contexts. Population-level analyses of kindergarten developmental outcomes reveal persistent inequities by ethnoracial background that are not fully explained by neighborhood income, underscoring the influence of structural and contextual factors beyond economic resources alone [10]. Related work demonstrates that multilingual kindergartners exhibit distinct profiles of developmental strengths and needs across multiple domains, highlighting the importance of holistic and culturally responsive approaches [11]. Further, studies linking kindergarten developmental health with neighborhood-level indicators reveal pronounced gradients in developmental vulnerability aligned with neighborhood opportunity and equity, illustrating how place-based conditions shape early health trajectories by age five [12]. Taken together, these findings position young children’s developmental health as a sentinel indicator of population well-being from a public health perspective. Monitoring early developmental outcomes at the population-level not only provides a snapshot of children’s current well-being, but also signals potential for future burdens of disease, chronic illness, and social disadvantage—as well as opportunities to foster healthier development, resilience, and thriving communities.

In recognition of this evidence, federal, state, and local governments have invested to varying degrees in early childhood programs such as early care and education, child tax credits, and nutrition assistance initiatives [13]. Yet, challenges in children’s developmental health persist [14–16], in part because policy approaches and political will needed to support development optimally during this sensitive life stage remain insufficiently mobilized. Also, most U.S. communities lack population-level data to assess whether policies are improving children’s developmental health.

Thus, monitoring early developmental outcomes at the population-level, alongside the evaluation of neighborhood conditions, is essential to assessing the impact of early childhood policies and programs, guiding resource allocation, and advancing equitable outcomes across communities [17]. This study examined how one state, New Mexico, has enacted an integrated early childhood policy framework and addressed this data gap by establishing a statewide, population-based system for measuring early development at kindergarten entry.

### Early childhood investments and developmental health in New Mexico

During the last few years, New Mexico has undertaken one of the most comprehensive early childhood policy reform efforts in the nation. In 2019, the state established the Early Childhood Education and Care Department, a cabinet-level agency consolidating programs across health, education, and family support sectors to improve coordination and accountability [18]. In 2020, the legislature established the Early Childhood Trust Fund to ensure stable, long-term financing for early learning initiatives. In 2022, voters approved the Land Grant Permanent Fund Constitutional Amendment, which together with the Trust Fund now provides more than $500 million annually in early childhood investments. Universal prekindergarten was expanded to all 3- and 4-year-olds in 2025. And after expanding child care assistance eligibility from 200% to 400% of the federal poverty level in 2022, New Mexico became the first state in the nation to establish a universal child care system in 2025, guaranteeing access to no-cost care for all families regardless of income [19]. Collectively, these policies represent an unprecedented and coordinated effort to strengthen the early childhood infrastructure, positioning New Mexico as an ideal context for examining developmental health and the population-level effects of systemic reform. This period reflects a critical transition, in which policies have been implemented but their full impacts have yet to be realized. 

New Mexico introduced these new policies in response to decades of data showing that the state’s children have not been achieving their full potential. Much of the current evidence, drawn from studies conducted before the new policies were introduced, is mixed. In a recent nationwide report on children of all ages (birth–18 years), the state ranked last in overall child well-being, with particularly low rankings in health (46th), economic well-being (49th), education (50th), and family and community context (50th) [20]. These rankings were calculated based on multiple child-relevant factors, including poverty, academic proficiency in reading and mathematics, child and teen mortality, and household characteristics such as parental education and family composition [20]. Additional data underscore these concerns: In 2023, approximately 25% of New Mexico children < 18 lived in poverty, compared with a national average of 16%, with the highest prevalence (27.1%) observed among children younger than 5 [21]. One-in-five children in the state has experienced two or more adverse childhood experiences, substantially higher than the national average of 14.5% [22]. Rates of child maltreatment are also elevated—12.4 per 1,000 children versus 7.4 per 1,000 children nationally [23]. Food insecurity compounds these risks, with New Mexico ranking 42nd in child food sufficiency in 2022–2023; only 61.8% of children lived in households that could afford nutritious food, compared with 67.3% nationally [24]. Educational outcomes are of particular concern in New Mexico, where state-level data for 2023–2024 showed that only 39% of students were proficient in reading and 23% in mathematics. New Mexico ranked last nationally among fourth graders in academic assessments.

Although New Mexico faces important challenges, a comprehensive view of early childhood developmental health also indicates areas of resilience and progress. Recent national studies using the National Survey of Children’s Health found that the proportion of children aged 3–5 years in New Mexico who were considered “healthy and ready to learn” did not differ significantly from national averages [25]. Data from the 2022–2023 survey further indicate that 67.8% of children are classified as “flourishing”—demonstrating curiosity, self-regulation, and resilience—placing the state sixth nationally on this measure [26]. Substantial state investments in early childhood education [27] also reflect progress: In 2025, New Mexico ranked sixth in preschool access for 3-year-olds, ranked fifth in state spending per child, and was among 18 states meeting at least 90% of the quality benchmarks established by the National Institute for Early Education Research [28]. Measures of poverty similarly indicate improvement. Although the official poverty measure places New Mexico’s child poverty rate at 27.4%, a supplemental poverty measure, which accounts for public assistance programs, reduces this estimate to 8.9%—below the national average [29]. This highlights the impact of recent anti-poverty policies [30, 31]. In 2024, New Mexico also expanded continuous Medicaid coverage for children through age 6, strengthening access to health care during the critical early years [32, 33] .

These paradoxical indicators make New Mexico an ideal natural environment for studying how early childhood policy reform may influence population-level developmental health outcomes.

### Study objectives

To assess kindergarteners’ developmental health amid significant early childhood policy reforms, New Mexico implemented a statewide, population-based assessment using the Early Development Instrument (EDI; *N* = 18,974). The EDI measures five domains—physical health and well-being, social competence, emotional maturity, language and cognitive development, and communication skills and general knowledge—providing a comprehensive picture of developmental health. By linking EDI data to the National Neighborhood Equity Index (NNEI), a standardized measure of local socioeconomic conditions [12], this study examined how developmental outcomes varied across demographic and neighborhood contexts.

Although some of New Mexico’s national child well-being rankings are concerning, many are based on broad indicators encompassing all children under 18 [34] and therefore, fail to capture stage-specific dimensions of development. By focusing on kindergarteners, this study provides a comprehensive assessment of developmental health during a critical period of growth and establishes a baseline for evaluating the population-level impact of the state’s early childhood policy reforms.

Thus, the study’s objectives were to (a) describe the statewide distribution of developmental health among New Mexico kindergarteners and (b) identify disparities across demographic and neighborhood contexts.

## Methods

### Study setting

This cross-sectional study was conducted in New Mexico between April and May 2024. New Mexico has a population of approximately 2.11 million, ranking 36th in the United States by population size [35]. In 2023, the state’s median family income was $62,268, below the national median of $80,610 [35] .

### Sample

This study used secondary data. Eligibility included kindergartners enrolled in New Mexico public schools during the study period. Private schools and Bureau of Indian Education schools were excluded; however, tribal members attending public schools were included. This cohort of children was born between 2018 and 2019 and turned three between 2021 and 2022, meaning they have potentially benefited from some but not all recent statewide early childhood policies.

### Data collection procedures

The current study used secondary data. In 2024, the University of California, Los Angeles (UCLA) Center for Healthier Children, Families, and Communitas (CHCFC) collected kindergarten data statewide on behalf of New Mexico. Specifically, UCLA CHCFC partnered with New Mexico’s Early Childhood Education and Care Department (ECECD) and the Public Education Department (PED) to coordinate the data collection. Caregivers received an informational letter with an option to opt out. With passive consent, kindergarten teachers received a one-hour training and orientation. Then they completed the EDI assessments, each of which took approximately 10–20 min and were administered at least 3 months into the school year to ensure sufficient familiarity with students. A total of 19,634 EDI records were collected, of which 660 (3.4%) were incomplete and deemed invalid. After exclusions, the final analytic sample consisted of *N* = 18,974 kindergarteners. This study uses those secondary data for the present analysis. Ethics approval was waived by the University of California, Los Angeles Institutional Review Board (IRB #25-2414).

### Measurements and instruments

#### Early development instrument

The EDI is a teacher-completed assessment with well-established psychometric properties across the United States, Canada, Australia, and other countries. Prior studies have demonstrated its robust multilevel validity, internal consistency, factor structure, and measurement invariance [36–39]. Kindergarten teachers completed the EDI for each student in their classroom, rating children across five domains: (a) physical health and well-being (13 items), (b) social competence (26 items), (c) emotional maturity (30 items), (d) language and cognitive development (26 items), and (e) communication skills and general knowledge (eight items). Children scoring above the 25th percentile were classified as “on-track,” those scoring above the 10th percentile but at or below the 25th percentile were considered as “at-risk,” and those at or below the 10th percentile were designated as “vulnerable.” The three-category domain scores were derived using established U.S.-wide normative percentile cutoffs rather than New Mexico–specific thresholds. These cutoffs were originally developed using an independent convenience sample of 10,244 kindergarten children in the United States during the 2009–2010 school year and have demonstrated predictive validity for later academic outcomes, providing a stable benchmark for examining developmental patterns and trends over time [37] .

The EDI also includes demographic indicators such as age, sex (male or female), and ethnorace (Hispanic/Latino/a; White; American Indian/Alaska Native; African American/Black; Asian, Native Hawaiian/other Pacific Islander; and other). All demographic variables were incorporated into the analytic models. In addition, the EDI determines whether a student has an individualized education program (IEP) and whether the student is an English language learner (ELL), thereby enabling a fuller understanding of developmental health and the potential need for specialized services.

#### National neighborhood equity index

To account for neighborhood-level socioeconomic context, the NNEI was also included. The NNEI is derived from U.S. Census American Community Survey 5-year estimates (2018–2022) and features 11 indicators across four dimensions: education, economic, wealth, and social factors. Neighborhoods are categorized into four levels of inequity: zero, low (1–2 barriers), medium (3–5 barriers), or high (> 6 barriers). Individual children were assigned NNEI values based on their home address geocoded to census tract. Importantly, previous research has demonstrated that NNEI levels predict EDI scores [12] .

### Analytic strategy

Descriptive statistics were used to summarize the dataset. The proportions of kindergartners classified as on-track, at-risk, or vulnerable were calculated for each EDI domain and a composite EDI score. EDI domain scores were analyzed as continuous variables (0–10 scale) in all regression models. Intraclass correlation coefficients (ICCs) were calculated to quantify the proportion of variance in EDI scores due to classroom level clustering. ICCs ranged from 14.1% to 27.3% across domains, indicating considerable between-classroom variance and appropriateness of multilevel modeling [40]. To account for the nested structure of the data (students within classrooms), multilevel regression models were estimated for each EDI domain using restricted maximum likelihood (REML) estimation. Models included random intercepts at the classroom level to account for clustering i.e., EDI surveys being completed by one teacher for multiple students in the same classroom. Fixed effects included sex, ethnorace, ELL & IEP status, and NNEI. Results are reported as regression coefficients with 95% confidence intervals and p-values (< 0.05, < 0.01, < 0.001). Sensitivity analyses were conducted comparing multilevel models to linear regression with and without cluster-robust standard errors; results were constant across all models (See supp 1). All analyses were conducted in Stata/MP 19.5 [41].

## Results

Only 132 (0.67%) of parents opted out of participation, and the ethnoracial composition of these children was comparable to that of the full study sample, with no differences observed in sex, ELL or IEP status. Data were drawn from 116 school districts and charters, encompassing 441 schools and 1,380 kindergarten classrooms. A total of 600 neighborhoods in New Mexico are represented. The analytic sample had an average age of 6.21years (*SD* = 0.33, range: 5.19–7.96). 51% of the children were male, with the remainder being female (49%). More than half identified as Hispanic/Latino/a (53%), followed by White (19%), American Indian/Alaska Native (9%), African American/Black (2%), Asian, Native Hawaiian/other Pacific Islander (1%), and other races/ethnicities (16%). Nearly 1 in 5 children were classified as ELL (18%), and a similar proportion had an IEP (18%). The NNEI further contextualized the distribution of children across neighborhood equity levels, with 61% living in neighborhoods with zero or low neighborhood equity barriers. Mean scores and standard deviation of EDI domains are shown. See Table 1 for more details.

**Table 1:** Sample descriptives, *N* = 18,974

	EDI Composite							
	Total		On Track		At Risk		Vulnerable	
Characteristic	*n*	%	*n*	%	*n*	%	*n*	%
*Sex*								
Female	9,288	49	5,143	55	2,297	25	1,848	20
Male	9,654	51	3,851	40	2,597	27	3,206	33
*Race and ethnicity*								
Hispanic, Latino/a	9,784	53	4,712	48	2,569	26	2,503	26
White	3,423	19	1,812	53	803	23	808	24
American Indian or Alaska Native	1,660	9	699	42	419	25	542	33
African American or Black	297	2	112	38	67	23	118	40
Asian, Native Hawaiian, or other Pacific Islander	238	1	147	62	51	21	40	17
Other	2,966	16	1,324	45	828	28	814	27
*English language learner*	3,485	18	1,248	36	998	29	1,239	36
*Individualized education program*	3,359	18	742	22	783	23	1,834	55
*National Neighborhood Equity Index*								
Level 0	4,312	23	2,316	54	1,040	24	956	22
Level 1 (low)	7,185	38	3,423	48	1,868	26	1,894	26
Level 2 (medium)	5,842	31	2,598	44	1,548	26	1,696	29
Level 3 (high)	1,635	9	671	41	449	27	515	31
*School information *								
School districts and charters	116							
Schools	441							
Classrooms	1,380							
*EDI Domain Score (0–10 scale)*, mean(SD)								
Physical Health & Wellbeing	8.6 (1.5)							
Social Competence	7.9 (2.1)							
Emotional Maturity	7.8 (1.8)							
Language & Cognitive Development	8.7 (1.9)							
Communication Skills & General Knowledge	7.0 (2.8)							

**Fig. 1:**
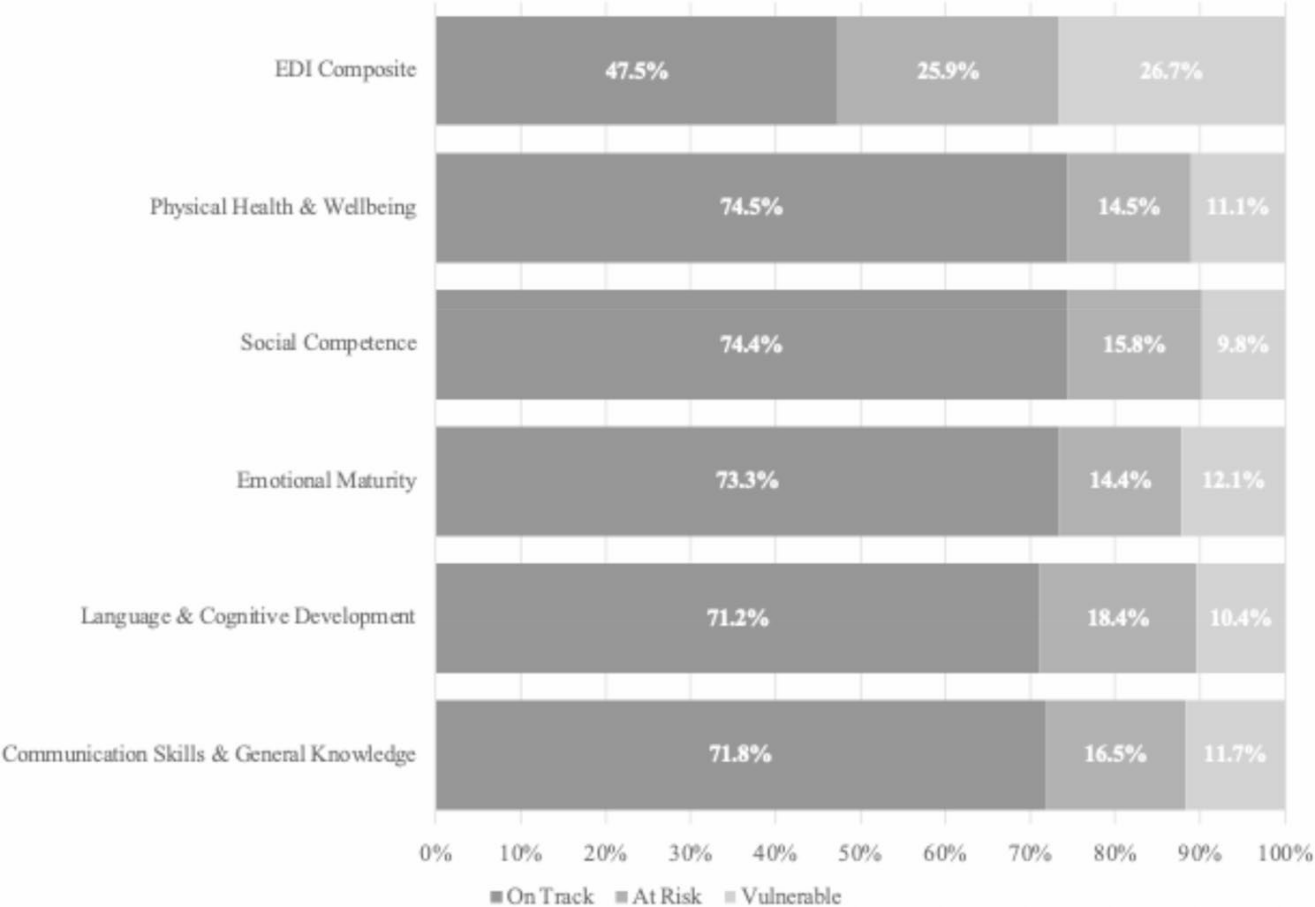
Statewide early development instrument composite and domain-specific scores in New Mexico

Figure 1 presents EDI composite scores, indicating that about 48% of children were classified as on-track, 26% as at-risk, and 27% as vulnerable. Domain-specific classifications revealed variation, with the proportion of children on-track ranging from 71.2% in language and cognitive development to 74.5% in physical health and well-being. Vulnerability was most pronounced in emotional maturity (12.1%) and communication skills and general knowledge (11.7%).

**Fig. 2:**
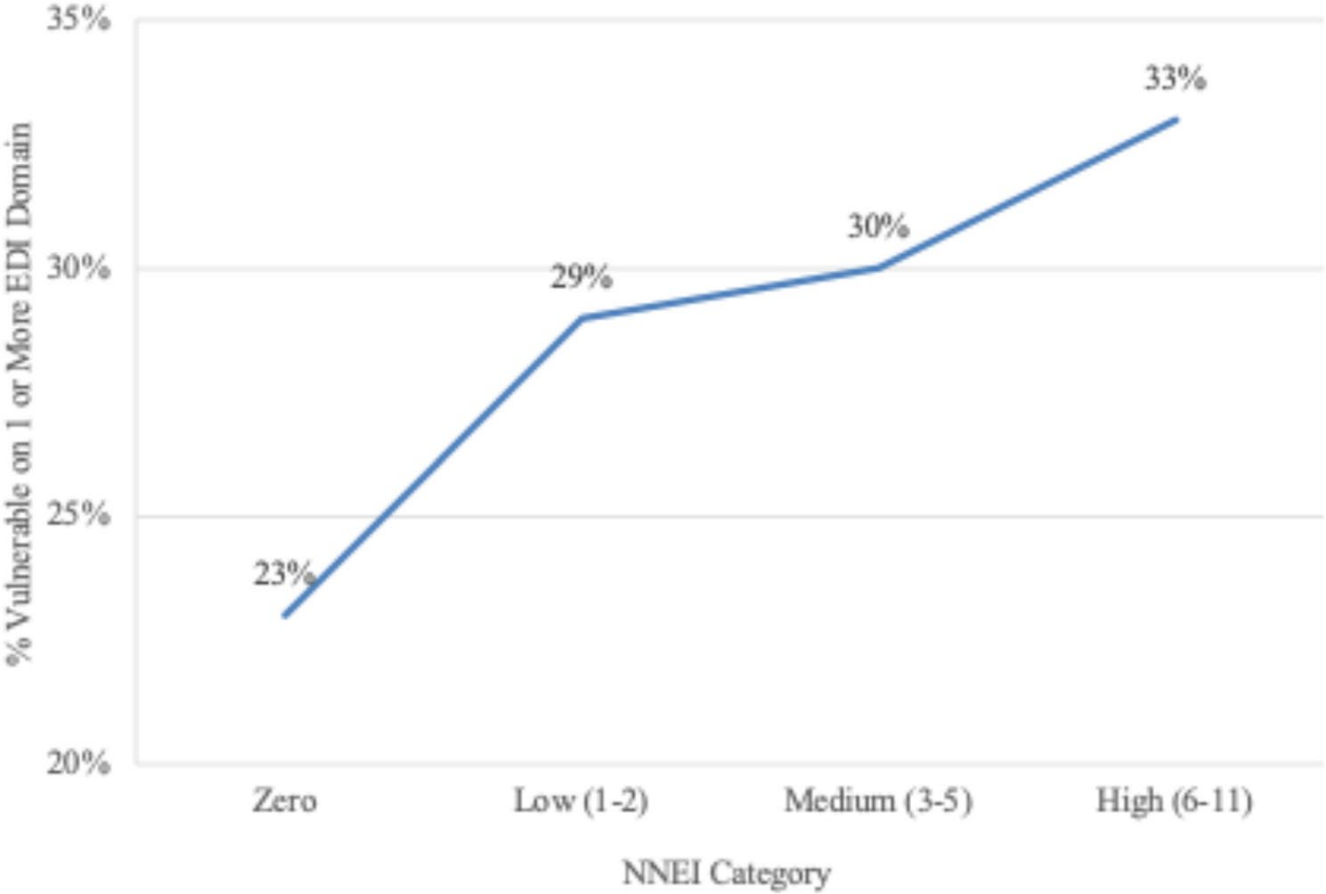
Percentage of children statewide in New Mexico (2024) vulnerable in one or more early development instrument domains by national neighborhood equity index level

Table 2 presents multilevel regression estimates for EDI domain outcomes, adjusted for demographics and covariates. Intraclass correlation coefficients ranged from 14.1% to 27.3% across domains, indicating substantial classroom-level clustering. Across all domains, male students scored significantly lower than female students. Ethnoracial disparities were also evident: African American/Black students scored lower across all domains compared to Hispanic/Latino/a students, while American Indian/Alaska Native students scored lower in language & cognitive development and communication skills. In contrast, Asian, Native Hawaiian/Pacific Islander and White students generally scored higher. ELL status was associated with lower scores across all EDI domains, with modest differences observed in social competence (coeff. = -0.2, 95% CI [-0.3, -0.2]) and progressively larger differences in language and cognitive development (coeff. = -0.8, 95% CI [-0.8, -0.7]) and communication skills and general knowledge (coeff. = -1.3, 95% CI [-1.4, -1.2]). These coefficients can be interpreted as average decreases in domain scores associated with ELL status, indicating small-to-moderate differences depending on the domain. In contrast, students with an IEP demonstrated substantially lower scores across all domains, especially in communication skills and general knowledge domain (coeff. = -2.5, 95% CI [-2.6, -2.5]), reflecting pronounced developmental disparities relative to their peers. Finally, children residing in neighborhoods with higher NNEI levels demonstrated significantly poorer outcomes across all domains, underscoring the relationship between neighborhood inequities on developmental health. Fig. 2 illustrates the proportion of children classified as vulnerable in one or more EDI domain by NNEI level. EDI vulnerability increased steadily with higher levels of inequitable neighborhoods, from 23% to 33%.

**Table 2:** Multi-level regression estimates for the early development instrument domain outcomes in New Mexico, adjusted for demographics and covariates, *N* = 18,974

	Physical Health & Well-Being		Social Competence		Emotional Maturity		Language & Cognitive Development		Communication Skills & General Knowledge	
	Coef	95% CI	Coef	95% CI	Coef	95% CI	Coef	95% CI	Coef	95% CI
Male sex	-0.2***	[-0.3, -0.2]	-0.7***	[-0.7, -0.6]	-0.8***	[-0.8, -0.7]	-0.2***	[-0.2, -0.1]	-0.6***	[-0.7, -0.5]
*Ethnoracial background*										
African American or Black	-0.3***	[-0.4, -0.1]	-0.6***	[-0.8, -0.4]	-0.6***	[-0.7, -0.4]	-0.2*	[-0.4, 0.0]	-0.4***	[-0.7, -0.2]
American Indian or Alaska Native	-0.1	[-0.2, 0.0]	-0.1	[-0.2, 0.1]	0.0	[-0.1, 0.1]	-0.2***	[-0.3, -0.1]	-0.7***	[-0.8, -0.5]
Asian, Native Hawaiian, or Pacific Islander	0.3***	[0.1, 0.5]	0.6***	[0.3, 0.8]	0.3***	[0.1, 0.5]	0.7***	[0.5, 0.9]	0.3*	[0.0, 0.6]
White	0.1**	[0.0, 0.1]	0.0	[0.0, 0.1]	0.0	[-0.1, 0.0]	0.2***	[0.2, 0.3]	0.4***	[0.3, 0.5]
Other	0.0	[-0.1, 0.1]	-0.1	[-0.2, 0.1]	0.0	[-0.1, 0.1]	-0.1	[-0.2, 0.1]	0.0	[-0.2, 0.1]
*English language learner*	-0.1***	[-0.2, -0.1]	-0.2***	[-0.3, -0.2]	-0.1**	[-0.1, 0.0]	-0.8***	[-0.8, -0.7]	-1.3***	[-1.4, -1.2]
*Individualized education program*	-0.8***	[-0.9, -0.8]	-1.3***	[-1.3, -1.2]	-1.0***	[-1.0, -0.9]	-1.3***	[-1.4, -1.2]	-2.5***	[-2.6, -2.5]
*National Neighborhood Equity Index*										
Level 1 (low)	-0.1***	[-0.2, 0.0]	-0.2***	[-0.2, -0.1]	-0.1***	[-0.2, -0.1]	-0.2***	[-0.2, -0.1]	-0.2***	[-0.3, -0.1]
Level 2 (medium)	-0.2***	[-0.2, -0.1]	-0.3***	[-0.3, -0.2]	-0.2***	[-0.3, -0.1]	-0.2***	[-0.3, -0.2]	-0.2***	[-0.3, -0.1]
Level 3 (high)	-0.2***	[-0.3, -0.1]	-0.2**	[-0.3, -0.1]	-0.1*	[-0.2, 0.0]	-0.3***	[-0.4, -0.2]	-0.3***	[-0.5, -0.2]
Observations	18,370		18,376		18,263		18,368		18,445	
ICC (Class-level)	27.3%		18.5%		23.6%		14.1%		23.2%	

Reference categories were female for sex, Hispanic or Latino/a for ethnoracial background, students who were not English language learners, students without an individualized education program, and Level 0 for National Neighborhood Equity Index

*ICC* Intraclass Correlation Coefficient

Models estimated using restricted maximum likelihood (REML) with random intercepts at classroom level

*p < .05. **p < .01. ***p < .001

## Discussion

This study represents the first statewide, population-level assessment of kindergarteners’ developmental health in New Mexico by linking EDI data with NNEI levels. Despite some of New Mexico’s low rankings on indicators of child well-being, findings from nearly 19,000 kindergartners show that the majority (> 70%) of children entered school developmentally on-track across distinct domains. However, only 47.5% of kindergarteners were on-track across all five domains collectively. Also, a clear social gradient emerged between neighborhood equity and developmental health. Developmental vulnerability was 10% points higher among children in high-barrier neighborhoods (33%) than in advantaged neighborhoods (23%). This gradient mirrors national analysis showing poorer outcomes with rising neighborhood disadvantage [12] and aligns with ethnoracial and income inequities observed in California [10]. Replication in a demographically distinct, largely rural state underscores the structural nature of developmental inequities.

Taken together, kindergarteners in New Mexico are doing as well or better than kindergarteners studied in other neighborhoods across the nation [14], a finding that is surprising considering the mixed child well-being statewide outcomes. Potential explanations for these observations must be considered. First, it is possible that if the EDI had been implemented historically across the state, kindergarteners may have always shown similar patterns of relative developmental resilience, only to falter educationally later when academic demands increased during higher grades. However, work in the United States and Canada showed that the EDI is in fact a good predictor of third- and sixth-grade educational outcomes [36–39], making this explanation unlikely. Second, it is possible that the comprehensive early childhood policy reforms put in place throughout New Mexico between 2019 and 2025 are starting to have an impact at a population-level, and when the kindergarten class of 2023–2024 reaches third and sixth grades, their educational outcomes will be better than previous cohorts. Although definitive conclusions cannot yet be drawn, the current findings offer cautious optimism.

Furthermore, New Mexico has used the EDI as a proximal indicator of early childhood well-being, rather than relying solely on later academic outcomes such as reading and mathematics performance in middle childhood. The state has also disseminated results at the neighborhood level, engaging professionals across disciplines and families through a network of community coalitions [42]. In this way, data are being used in real time not only to look back on the impact of policy, but also to alert local leadership to current patterns of developmental needs in the community that must be addressed. These data then inform community-based co-development of interventions to improve targeted aspects of young children’s development—e.g., language and cognitive development—that are tailored to local conditions. This work is especially important in neighborhoods with most disadvantage, where there is a concentration of children with developmental vulnerability. Local governments might then choose to direct additional early support to improve these local developmental ecosystems, giving young children the best chance of a good start in life.

Kindergarteners in the cohort showed highest rates of vulnerability on the EDI emotional maturity domain, which assesses ability to regulate behavior and emotions, especially in the areas of pro-social behavior, empathy, anxiety and aggression, concentration and impulse control [43]. Emotional development is now understood to be closely intertwined with academic learning in the early years, and skills in this domain set the foundation for positive life outcomes. For young children their environments, both physical and psychological; opportunities for play; and the quality of their relationships all impact their emotional maturation [44, 45]. This finding of population-level vulnerability creates an opportunity to prioritize enhanced support for emotional development both in early years and in school settings, for example through guided participation in play [46] in ways that model and apply healthy emotional skills, and explicit teaching of skills e.g. guiding children in initial appraisal of events and their emotional meaning, awareness of their emotional response and its regulation followed by pro-social action [47, 48]. Improvements in emotional development in these early years could have a large impact on health trajectories across the life course.

Beyond EDI domains, different demographic patterns of developmental challenges emerged. African American/ Black children, English Language Learners and students with Individualized Education Programs scored lower across all domains, suggesting a need for comprehensive, cross-domain developmental supports. These findings highlight the importance of tailoring interventions to reflect cultural diversity and varied developmental needs. Such supports may include multilevel strategies, such as increased access to adaptive play environments, high-quality language enrichment across home and childcare settings, and community-based programs that promote social-emotional development within local neighborhoods. Importantly, these resources should be culturally affirming – centering meaning, identity, and belonging – while remaining responsive to the unique needs of diverse populations. These results also underscore the interconnected nature of early childhood development. Challenges in one domain (e.g. language development) may contribute difficulties in others (e.g. social skills) [49], and co-occurring challenges across multiple domains may compound over time, ultimately affecting overall functional ability.

In contrast American Indian/Alaska Native students scored lower only in language & cognitive development and communication skills, showing relative resilience in social, emotional, and physical development. While the reasons for these patterns are beyond the scope of this paper, it is possible that the Native American communities deeply established history in the region coupled with cultural supports from one of the largest Indigenous populations in the US and strong intergenerational links are protective for aspects of these students development [50, 51]. Supports should build on these strengths and prioritize culturally resonant language and cognitive enrichment that are co-developed with members of the Native American community.

### Limitations

The study has several limitations. First, although about 80% of kindergarteners in the state were captured in the study, children attending private or Bureau of Indian Education schools or who were homeschooled were not included. Therefore, it is unclear whether their EDI findings would have aligned with or differed from those of children attending public school. Second, the EDI relies on teacher report rather than objective testing; however, prior studies have confirmed its high validity and predictive educational value with no significant bias [30–33], giving us confidence in the measure. Third, the measure does not include parents’ evaluation of their children’s development or information on key early exposures (e.g., child care or preschool attendance). The forthcoming inclusion of parent-reported data through the Child Health Experiences Questionnaire will enrich interpretation by incorporating family perspectives and prior experiences that may influence kindergarten readiness and developmental health. Fourth, the data only reflect findings for one kindergarten year, and it is possible that this year may have been unusual in some way. This kindergarten cohort was born in 2018–2019 so their early years were impacted by the Covid-19 pandemic which had secondary effects on increased time spent in the home environment, childcare closures and levels of family stress, all of which may have affected these results. While these factors may have influenced the proportion of children vulnerable in emotional maturity, as has been suggested in other studies [52,53] only future serial EDI measures will be able to determine trends.

## Conclusions

This study offers insights applicable across national efforts aimed at monitoring early childhood population health amid early childhood policy innovation. The findings underscore the importance of establishing a clear baseline during policy reform by pairing a population-based measure of early childhood developmental health (e.g., EDI) with a neighborhood-level measure (e.g., NNEI) to contextual results and identify communities most in need of support. Population-based tools enable policymakers to track progress, guide investments, and strengthen accountability for the outcome that matters most—the healthy development of young children. In New Mexico, continued monitoring of successive kindergarten cohorts will be essential to determine whether children exposed to the full implementation of early childhood policy reforms experience sustained improvements over time. Longitudinal tracking will further reveal whether these policy innovations translate into lasting gains in health, education, and overall well-being for future generations.

## Data Availability

The data used for the current study are not publicly available due to contractual obligations. For any questions, the first author should be contacted: Dr. Judith Perrigo at jperrigo@luskin.ucla.edu.
